# Connective Tissue Fibroblast Properties Are Position-Dependent during Mouse Digit Tip Regeneration

**DOI:** 10.1371/journal.pone.0054764

**Published:** 2013-01-18

**Authors:** Yuanyuan Wu, Karen Wang, Adrine Karapetyan, Warnakulusuriya Akash Fernando, Jennifer Simkin, Manjong Han, Elizabeth L. Rugg, Ken Muneoka

**Affiliations:** 1 Department of Cell and Molecular Biology, Tulane University, New Orleans, Louisiana, United States of America; 2 Department of Dermatology, University of California Irvine, Irvine, California, United States of America; University of Minnesota Medical School, United States of America

## Abstract

A key factor that contributes to the regenerative ability of regeneration-competent animals such as the salamander is their use of innate positional cues that guide the regeneration process. The limbs of mammals has severe regenerative limitations, however the distal most portion of the terminal phalange is regeneration competent. This regenerative ability of the adult mouse digit is level dependent: amputation through the distal half of the terminal phalanx (P3) leads to successful regeneration, whereas amputation through a more proximal location, e.g. the subterminal phalangeal element (P2), fails to regenerate. Do the connective tissue cells of the mammalian digit play a role similar to that of the salamander limb in controlling the regenerative response? To begin to address this question, we isolated and cultured cells of the connective tissue surrounding the phalangeal bones of regeneration competent (P3) and incompetent (P2) levels. Despite their close proximity and localization, these cells show very distinctive profiles when characterized in vitro and in vivo. In vitro studies comparing their proliferation and position-specific interactions reveal that cells isolated from the P3 and P2 are both capable of organizing and differentiating epithelial progenitors, but with different outcomes. The difference in interactions are further characterized with three-dimension cultures, in which P3 regenerative cells are shown to lack a contractile response that is seen in other fibroblast cultures, including the P2 cultures. In in vivo engraftment studies, the difference between these two cell lines is made more apparent. While both P2 and P3 cells participated in the regeneration of the terminal phalanx, their survival and proliferative indices were distinct, thus suggesting a key difference in their ability to interact within a regeneration permissive environment. These studies are the first to demonstrate distinct positional characteristics of connective tissue cells that are associated with their regenerative capabilities.

## Introduction

The emerging field of regenerative medicine encompasses multiple disciplines including tissue engineering, stem cell biology, and regenerative biology [1]. The field of regenerative biology is the study of a biological systems' ability and limitation to re-new itself and the underlying mechanisms that were applied by this system. A better understanding of regenerative biology would correlate to new approaches to tissue engineering and stem cell biology and link directly to new clinical approaches in regenerative medicine.

The regenerating salamander limb has long been an accepted model for epimorphic regeneration, and the mechanism for this response appears to be a relationship between stem-like cells and their ability to form a blastema [2], [3]. In studies of the salamander blastema, the cellular contribution appears to be derived primarily from fibroblasts that migrate into, and dedifferentiate within the amputation wound. [4], [5], [6], [7], [8]. Bryant et al (2002) has proposed that fibroblasts in amphibians are a quiescent stem cell population that can be activated upon tissue injury. Presumably this activation of fibroblasts must involve a dedifferentiation or reprogramming response that up-regulates cell cycle genes, activates the cytoskeleton for cell migration, and initiates the re-expression of embryonic genes important for limb development. Fibroblasts, present throughout the mammalian body and shown to be re-programmable in vitro [9], represent a large potential cell population for regeneration. However, unlike amphibian fibroblasts, the involvement of mammalian fibroblasts in injury responses is not generally associated with a regenerative response, but with a fibrotic response that culminates in scar formation [10]. The importance of fibroblasts in amphibian limb regeneration, combined with the role that fibroblast play in scar tissue formation in mammals, has led to the conclusion that their response to injury is key to distinguishing between a regenerative versus a wound healing response [11].

In recent years the murine regenerating digit tip has become an important mammalian model for regeneration. The regeneration of amputated distal digit tips has been reported in both rodents and primates, including humans [12], [13], [14], [15] and in adult as well as developing tissues [1]. The adult mouse digit tip can undergo a successful regeneration response that, like the neonatal digit tip, involves the formation of a blastema [16], [17]. This regeneration response is level dependent, amputation through the mid-region of the terminal phalangeal element (P3) regenerates, whereas amputation through the proximal P3 region fails to regenerate [16], [18]. In the failed regeneration responses of the sub-terminal phalangeal element (P2) and proximal P3amputations, a wound healing response that involves wound contraction with the formation of scar tissue occurs [19]. The level-dependent regeneration of the mouse digit provides a model in which relatively similar amputation wounds produce very different responses, i.e. regeneration versus scar formation.

We focused on fibroblast cells derived from the connective tissue for two main reasons: (1) these cells play a key role in amphibian limb regeneration as well as mammalian wound healing [11], [20], and (2) fibroblasts have been implicated as the cell type in the human body that maintains position information [21], [22]. To address the connective tissue contributions, we established fibroblast cell lines from primary cultures of cells derived from the regeneration competent P3 region and from the neighboring, regeneration incompetent P2 region of the adult mouse digit. These cell lines were characterized in vitro and in vivo, and our results indicate that they are best described as fibroblast progenitor cells. P2 and P3 cells were similar in morphology, doubling time, and immunophenotype; however both cell lines possess and maintain distinctive position-specific characteristics even during expansion. In three-dimensional skin equivalence cultures, P2 cells induced keratinocytes to differentiate into stratified epidermis, whereas P3 cells induced the aggregation of keratinocytes to form structures with a nail-like morphology. Despite these differences, in vivo studies demonstrate that both P3 and P2 cells can participate in blastema formation and digit tip regeneration, or in wound healing associated with scar formation. Thus, P2 cells are regeneration-competent even though they are derived from a regeneration-incompetent region of the digit. These studies provide evidence that regeneration-competent cells are present at non-regenerating wound sites, and that the wound environment itself is able to direct regeneration-competent cells either toward a regenerative event or a wound healing event.

## Materials and Methods

### Amputations and animal handling

Mouse strains used in this study included outbred CD1 purchased from Charles River Inc., and C57BL/6-Tg(ACTB-EGFP)1Osb/J (EGFP), B6;129S-*Gt(ROSA)26Sor*/J (ROSA26) and NOD. CB17-*Prkdc^scid^*/J (SCID-NOD) mice purchased from Jackson lab. By crossing C57BL/6-Tg(ACTB-EGFP)1Osb/J mice with B6;129S-*Gt(ROSA)26Sor*/J mice we established a colony of mice expressing both *LacZ* and *eGFP*. These mice were used to generate labeled cells for our cell marking studies. SCID-NOD mice were used as host for cell marking studies to minimize a rejection response. Initial studies established P2 and P3 cell lines from CD1 mice and subsequent lines were established from marked strains in an identical manner. Hindlimb digits were used for all amputations. All experiments were performed in accordance and approval of the Institute Animal Care & Use Committee of Tulane University Health Science Center.

### Isolation and expansion of position-specific connective tissue cells

**Figure 1:**
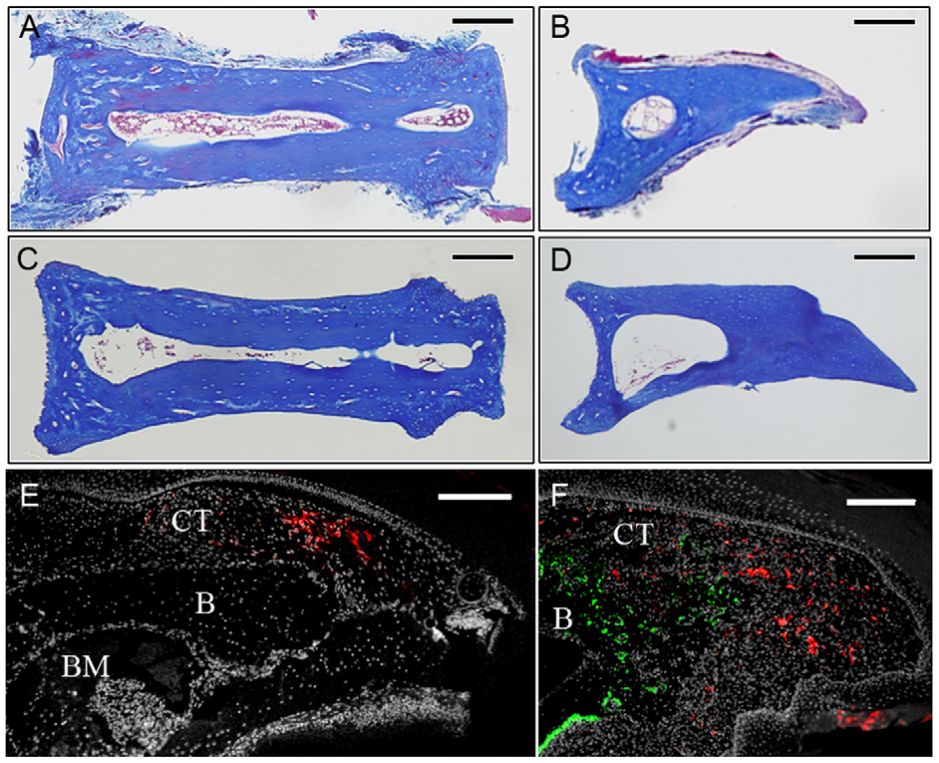
Isolation of connective tissue cells from the P2 and P3 regions. A, B) Histological section of the P2 (A) and P3 (B) bone after dissection. Loose connective tissue is attached to the surface of the intact bone. C, D) After enzymatic digestion the majority of loose connective tissue was digested off the bone while tissues within the bone marrow are still present. E) DiI labeled cells (red) within the connective tissue (CT) dorsal to the P3 skeletal element (B) are clustered in the amputation stump 2 days post-injection. BM, bone marrow. F) Blastema stage regenerate at 13 DPA showing DiI labeled cells (red) scattered throughout the blastema but not overlapping with Osteocalcin immunohistochemical labeling (green) to identify regenerating osteoblasts. The blastema is contiguous with the connective tissue (CT) and bone (B) of the stump. Scale bars  = 200 µm.

To establish P2 and P3 connective tissue fibroblast lines, cells were isolated from adult female mice 7–8 weeks old. All digits from both hindlimb and forelimbs were collected in dissection media (DMEM high glucose, 2 mM glutamine, 0.5 mg/ml gentamycin, 2% FBS; Gibco) and manually dissected. Skeletal elements were isolated away from skin, fat pad and nail, and P2 and P3 phalangeal elements were isolated by separating the joints with a sharp needle. P2 and P3 phalangeal elements were sorted and treated separately. Histological analyses of P2 and P3 elements showed connective tissue associated with the skeletal elements (Figure 1A, B). P2 or P3 elements were transferred into dissection medium supplemented with 1.0 Wunsch unit/ml liberase blendzyme I (Roche applied science, catalogue#11988409001) and incubated overnight at 37°C in a tissue culture incubator (5% CO_2_). Skeletal elements were removed, cells were pelleted, re-suspended in mesenchymal stem cell medium [23] and plated on fibronectin coated dishes. During expansion the media was changed every 3–4 days. Histological analyses of the skeletal elements following treatment demonstrated the successful removal of all adherent connective tissue (Figure 1C, D).

### Flow cytometry/FACS

The antibodies used for immunophenotyping by flow cytometry included Sca-1, CD29, V-CAM, CD34, CD104, CD45, CD49f (Biolegend), and C-kit (BD biosciences). Cells were trypsinized, centrifuged, washed twice and re-suspended in PBS. Suspended cells were treated with antibodies for 40 min and after 3 washes in PBS analyzed with a Becton-Dickinson FACSVantage SE cell sorter to detect reactivity. Each antibody was tested individually and with isotype controls.

### RT-PCR

Total RNA from each cell line were isolated using TRIZOL reagent (Invitrogen), and for cDNA synthesis we used SuperScript® III First-Strand Synthesis System (Invitrogen). Primers used for RT-PCR are as follows: GAPDH [24], BMP4-F 5′-TATGAAGCCCCCAGCAGAAA TG-3′, BMP4-R5′-ATAGTGAATGGCGACGGCAGTT-3′ Msx1-F 5′-CCTCTCGGCCATTTCTCAG TCG-3′, Msx1-R 5′ -GGGTTTCGCGGCCATCTTCAG-3′, Oct4-F 5′-TGTCCGCCCGCATAC GAGTTCT-3′, Oct4-R 5′-GCAGGGGCCGCAGCTTACACAT-3′, Rex1-F 5′-GAAGAAAACG GCAAAGACAAGTGG-3′, Rex1-R 5′-GGGCCGCCTGCAAGTAATGA-3′, Sox2-F 5′-ACGC AAAAACCGT GATGCCGAC-3′, and Sox2-R 5′-CGTTTGCCTTAAACAAGACCACG-3′. All results were normalized to GAPDH.

### In vitro keratinocytes-fibroblasts co-culture assay

Skin equivalents were prepared in either 6 or 12 well cell culture (Thinsert 3 µm pores; Greiner Bio One) as described [25]. Collagen gels were prepared from collagen type I (BD Biosciences) seeded with 2.5×10^4^ cells/ml and incubated in mesenchymal stem cell medium at 37°C in 95% air/5% CO_2_ in a humidified incubator. 2.0×10^5^ neonatal human epidermal keratinocytes (nHEK; Cascade Biologics, Portland, OR) were seeded on to the surface of the gel and incubated for 1 hour to allow cell attachment. The gels were submerged in Epilife medium supplemented with Epilife defined growth supplement (Cascade Biologics, Portland, OR) and 1.82 mM CaCl_2_. They were incubated for 2 days after which time the medium was adjusted such that the keratinocytes were at the air/liquid interface. The cultures were incubated under these conditions for up to 3 weeks with medium changes every second or third day. For histological analyses, gels were washed with PBS and incubated for 4h in 30% sucrose at 4°C. The gel was removed from the insert using spatula and placed cryomold with OCT compound (Sakura Finetek USA Inc, Torrance, CA) and incubated for 30 min at room temperature before freezing in liquid nitrogen vapor. 10 µm cryosections were cut and stained with hematoxylin and eosin (Biomeda) according to the supplier's protocol. For whole mount analyses the gels were washed with Dulbecco's phosphate buffered saline (DPBS), fixed with 10% formalin for 30 minutes, followed by washing with DPBS. Gels were incubated in Rhodanile blue, which is composed of 0.1% Nile Blue A (Sigma) and 0.1% Rhodamine B (Sigma) in water, for 1 to 2 minutes and washed with tap water. For gel contraction studies, collagen gels were seeded with a variable number of P2 or P3 cells. The gels were released from the side of the insert using a pipette tip and were allowed to contract for a further five days. For the effect of keratinocytes on gel contraction, skin equivalent cultures were released from the side of the insert and allowed to contract for five days under submerged conditions.

### Cell marking studies

For in vivo cell marking, injection of DiI (CellTracker^TM^ CM-DiI, Invitrogen; 0.1 mg/ml in 0.3 M sucrose,1% ethanol, and 0.05% Nile blue sulfate,) was performed using pulled glass micropipettes and a microinjector as previously described [26], [27]. The DiI solution was injected into the dorsal connective tissue between the nail and the P3 element at 3 days post-amputation (DPA) to minimize non-specific labeling associated with enhanced cell necrosis following amputation injury. Injected digits were observed using a fluorescence dissection microscope (Leica MZFL*111*) and the extent of the injection site was validated histologically. Digits were collected for analysis at 12–13 days post-amputation.

For in vivo cell injection, cells were concentrated in culture medium at approximately 3.75×10^7^ cells/ml and mixed with an equal volume of hydrogel (BD™ PuraMatrix™ Peptide Hydrogel). Trypan blue was added to the cell suspension to aid in visualizing injections. The cell suspension was maintained on ice until injection. Adult SCID-NOD mice were used as hosts and injections targeted either digit 2 or digit 4 of the hindlimb. Mice were anesthetized with Nembutal sodium solution as described [24]. Injection into the P3 region targeted the dorsal connective tissue between the nail and the P3 skeletal element. Injection into the P2 region targeted the region between the dorsal skin and P2 bone element. One day after injection, the digit was amputated midway through the phalangeal element. Regenerating P3 tissues were collected at 10 and 16 DPA and non-regenerating P2 amputated tissues were collected at 16 DPA. Digit tissue was snap-frozen, cryoshaved, fixed with 0.25% glutaraldehyde and stained with X-GAL staining solution (40–50 mg/ml X-gal substrate, Promega V3941). After X-GAL staining the tissues were fixed in Z-fix (Anatech LTD, 174) overnight at room temperature (RT) and processed for paraffin histology. For control cell injections, we used a GFP labeled human breast cancer cell line (M4A4 LM3-4CL16GFP) purchased from ATCC (CRL-2917™). Digit tissue was harvested, fixed with Z-fix overnight at RT and processed for paraffin histology.

### Immunohistochemistry

Labeled cells were identified based on immunohistochemical staining with a GFP antibody (Novus biologicals). The differentiation of M4A4 LM3-4CL16GFP cells was evaluated based on immunohistochemical staining with Von Willebrand factor VIII (VWF) (Dako) using Proteinase K epitope retrieval. To identify osteoblast differentiation, immunohistochemistry was performed using osteocalcin (OCN) (Takara) with proteinase K epitope retrieval. For proliferation and cell death evaluation, immunohistochemical staining for Ki67 and cleaved caspase 3 (Biocare Medical) was performed using heat epitope retrieval. All immunohistochemical stains used AlexFluor conjugated (Invitrogen Molecular Probes) secondary antibodies, combined with nuclear DAPI stain (Invitrogen).

## Results

### Connective tissue cells contribute to blastema formation and digit tip regeneration

No definitive technique exists to genetically trace fibroblast cell populations. To better characterize the involvement of connective tissue cells in the regeneration response we labeled cells by localized microinjection using the lipophilic cell marker DiI. The connective tissue surrounding the terminal phalanx bone consists of multiple cell types including fibroblasts, endothelial cells, vascular smooth muscle cells and pericytes, thus this labeling strategy does not distinguish the relative contribution from these different cell types to the regenerate. We introduced DiI into the dorsal region of a distally amputated digit after the wound had stabilized, (3 DPA) and prior to wound closure [28]. Two days after DiI labeling a discreet and contiguous region of the stump dermis was DiI labeled (Fig. 1E). When blastemas were analyzed at 12–13 DPA, individual DiI labeled cells were found scattered throughout the blastema (Fig. 1F), with only a small percentage of DiI labeled cells present at the injection site. The 12–13 DPA blastema initiates osteogenesis based on the presence of Osteocalcin expressing osteoblasts at the stump interface, and we find no co-localization of DiI labeled connective tissue cells with osteoblasts (Fig. 1F). These data demonstrate the participation of connective tissue cells in the formation of the regenerative blastema. The scattered distribution of labeled cells in the blastema is suggestive of a role for cell migration during the healing response, and the absence of double labeled cells is consistent with previous studies suggesting that many cell types are lineage restricted during the regeneration process [29], [30].

### Connective tissue cell lines

To begin to explore the role of connective tissue cells in the regenerative response, we began by isolating cells from the connective tissue of the regeneration competent terminal phalanx (P3) and the non-regeneration competent sub-terminal phalange (P2). Using outbred CD1 female mice, connective tissue associated with the P2 and P3 bones were isolated by dissection. Cells from these P2 and P3 bones with connective tissue were released from the extracellular matrix via enzymatic digestion and selectively expanded in MSC culture medium. To be assured of cell isolation and line consistency and reproducibility, cells were isolated (n>4) and expanded in multiple animals. During the initial expansion, we documented cell number after each passage and based on these data we were able to determine the average doubling time. The average doubling time for both P2 and P3 cells increased during the first 4 passages, an indicator of in vitro selection, and eventually stabilized at 35.5 and 29.5 hours, respectively. P2 and P3 cells have been maintained in continuous culture for an extended period (>5 months; >22 passages), successfully frozen and thawed, and re-established in other labs; thus these cells represent stable lines.

**Figure 2:**
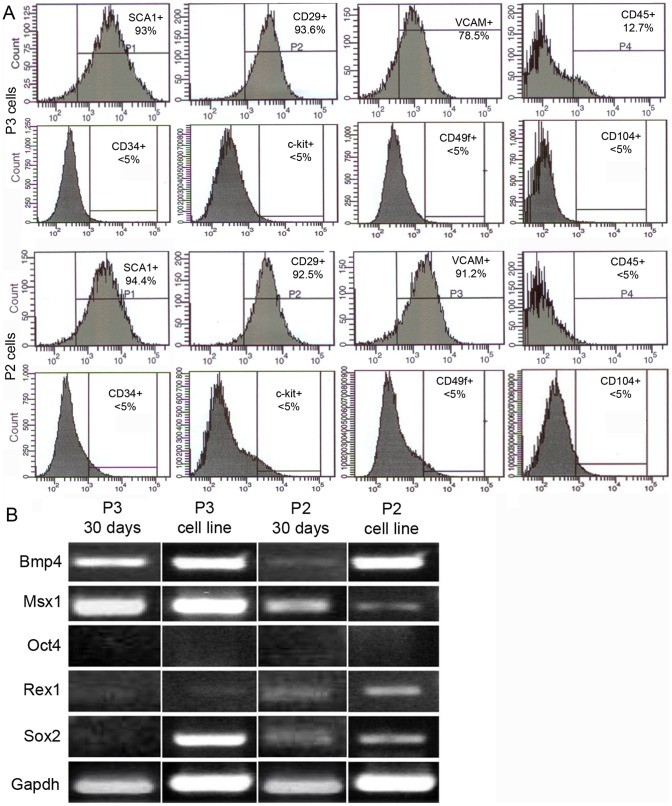
Flow cytometric profiling and RT-PCR analysis of P2 and P3 cells. A) Flow cytometry analysis identify a similar signature for P2 (bottom panels) and P3 cells (top panels). Analyses were performed in series on the same samples, P1-P4 denote the order of the measurements. In the scatter plots, both cell lines are positive with a single peak for Sca-1, CD29, V-CAM and negative for CD45, C-kit, CD49f, CD104 and CD34. This signature is similar to other characterized MSC lines. B) RT-PCR results from both P3 and P2 cells expanded for 30 days compared with established cell lines. Digit regeneration marker genes *Bmp4* and *Msx1* are stably expressed in P3 cells and variably expressed in P2 cells. Stem cell marker genes are differentially expressed in P2 and P3 cells. *Oct4* is not expressed in P2 and P3 cells. *Rex1* is minimally expressed in P2 and P3 cells. *Sox2* is minimally expressed in P2 cells and in early passage P3 cells but strongly expressed in the P3 cell line.

P2 and P3 cells were initially characterized based on an analysis of expressed stem cell surface markers. We tested both early passage cells (passage 3–4) and stable lines, characterizing immunoreactivity to known surface markers using flow cytometry [23], [31], [32]. Early passage and stable lines displayed similar profiles. Both early and stable lines of P2 and P3 were positive for Sca-1, CD29, and V-CAM; negative for CD45, C-kit, CD49f, CD104 and CD34 (Fig. 2A). The absence of hematopoietic stem cell surface markers: CD45, C-kit, and CD34 [33], [34] and keratinocyte progenitors (CD104) [35], [36] supports the isolation of a mesenchymal fibroblast cell population not associated with circulating cells and epidermis. This profile is similar to established marrow-derived MSCs [22], [28].

Early passage and stable lines were characterized using semi-quantitative RT-PCR to establish whether stem cell marker genes and known digit tip regeneration marker genes were expressed (Figure 2B). The embryonic stem cell marker *Oct4* was not expressed in either early passage or stable lines of both P2 and P3 cells, thus distinguishing them from other described MSC lines [23], [37]. Two stem cell genes (*Rex1* and *Sox2*) previously linked to a digit amputation response in mice [38] were differentially expressed in P2 and P3 cells. *Rex1* was expressed at a low level at early and late passage stages in both P3 and P2 cells. *Sox2* was not detected in early passage P3 cells but was expressed in P3 cell lines, whereas P2 cells displayed stable low level expression throughout. *Bmp4* and *Msx1* have been shown to be functionally required for digit regeneration in mice [24], [39], [40]. *Bmp4* and *Msx1* transcripts are abundant in both early passage and late passage P3 cells. In P2 cells, *Bmp4* is expressed at a low level during early passage but is abundant after expansion, whereas *Msx1* transcripts display a reciprocal pattern. In general, using a small number of developmental and stem cell markers, the expression profiles between early and late passage cells P2 and P3 cells identify them as uniquely different from each other and from previously characterized stem cells.

### Organotypic cultures show P2 and P3 cells maintain position-specific characteristics

**Figure 3:**
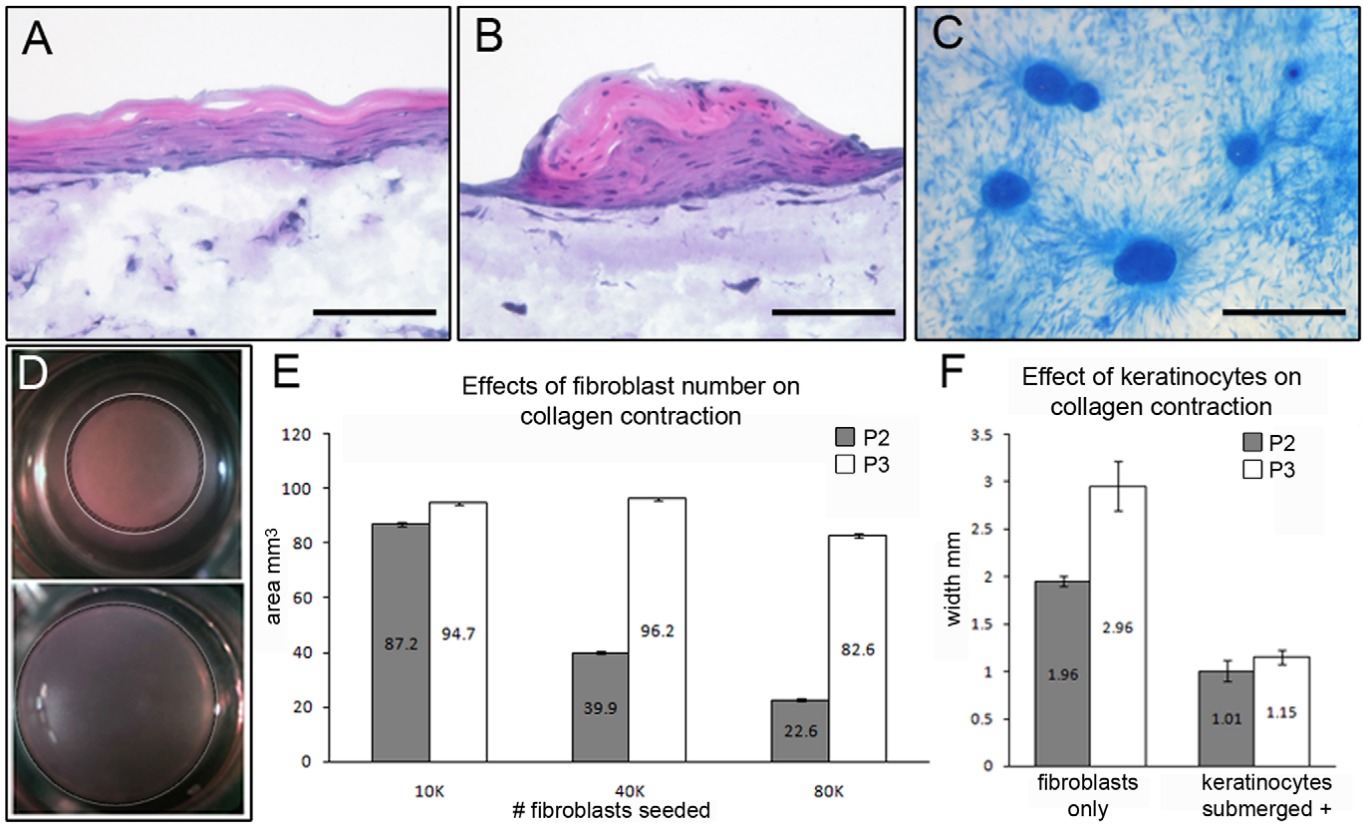
Position-specific characterization of P2 and P3 cells. A) Hematoxylin and eosin stained sections of skin equivalents generated from P2 cells co-cultured with human keratinocytes showing the induction of stratified sheets of differentiating epidermis. B) Hematoxylin and eosin stained sections of skin equivalents generated from P3 cells co-cultured with human keratinocytes showing the induction of aggregate structures that display keratosis similar to that observed from cultures of nail matrix epidermis. C) Whole mount preparations of P3 skin equivalents stained with Rhodanile blue showing the aggregation of keratinocytes induced by P3 cells. D) Collagen gels seeded with P2 cells (top) resulted in a contracted gel phenotypes whereas gels seeded with P3 cells did not display a contraction response. E) Area measurements of collagen gels show that P2 cells induced a cell density dependent contraction response, whereas P3 cells failed to contract the gel at similar seeding densities. F) Co-cultures of P2 cells with human keratinocytes enhanced the contraction response which was measured by determining the width of the collagen gel. Keratinocyte co-cultured with P3 cells induced a contraction response that was of a similar magnitude. All chart measurements are mean ± SEM (n = 3). A and B, scale bar  = 100 µm; C, scale bar  = 300 µm.

In mammalian epimorphic regeneration, the mesenchymal connective tissue component must be replaced, however, other cell lineages, specifically the ectoderm, must also be replaced. The feedback signaling mechanisms that exist between the ectoderm and mesoderm lineages are necessary in developmental and regenerative models. To address if our new lines interact differently with the ectoderm lineage, we employed three-dimensional organotypic cultures that simulate *in vivo* growth conditions and provide a useful model for probing epithelial-mesenchymal interaction [41]. In skin equivalent cultures containing fibroblasts covered by progenitor keratinocytes, fibroblasts direct keratinocyte differentiation [42]. Keratinocytes cultured with P2 cells formed epithelial sheets which differentiated a typical layered epidermis and showed signs of cornification (Figure 3A). This phenotype is typical of that observed when keratinocytes are co-cultured with primary human fibroblast or mouse fibroblast cell lines [43], [44]. In contrast, the same keratinocytes cultured with P3 cells formed aggregate structures that displayed keratosis (Fig. 3B, C) similar to that observed from cultures of nail matrix epidermis [45]. The epidermal aggregates show signs of differentiation but nuclei persisted in the upper cell layers (parakeratosis) and there was a lack of any obvious stratum corneum. These features are more characteristic of mucosal epithelia than the epidermis [46]. P2 cells were derived from a region of the digit that is encased by skin whereas P3 cells are derived from the digit tip which contains the nail organ. These results reveal P2 and P3 cells maintain distinctive position-specific interactions with keratinocyte progenitors seen in their aggregation in the P3 co-cultures, and the organized sheets of keratinocytes in the P2 co-cultures.

To better understand these distinctive physical interactions, we cultured P2 and P3 cell lines in collagen gels. Fibroblasts cultured in collagen gels typically cause the gel to contract, a characteristic associated with a fibrotic response in vivo [47]. Collagen gels prepared with P2 cells displayed the expected gel contraction response (Fig. 3D). The degree of collagen gel contraction by P2 cells was cell number dependent (Fig. 3E) in much the same way that human dermal fibroblasts contract gels when similarly cultured [48], [49]. When collagen gels were prepared with P3 cells, we failed to see a similar contraction response (Fig. 3D), and increasing the initial density of P3 cells had no effect on gel contraction (Fig. 3E). However, contraction of P3 cells seeded gels was induced by the addition of keratinocytes; after two days of growth in the presence of keratinocytes the amount of gel contraction was similar in both P2 and P3 seeded cultures (Fig. 3F). These results show that P2 and P3 cells display fibroblast-like characteristics but that these cells are distinct in their contractile capabilities, and that P3 cells are responsive to epithelial signals.

With respect to regenerative capabilities, connective tissue cells are expected to possess a number of characteristics that are demonstrated by our in vitro studies. Both P2 and P3 cell lines are similarly proliferative and display stem cell characteristics based on immunophenotyping. Yet, in vitro three dimensional assays and epidermal co-cultures show that P3 cells are inimitable and distinctive by comparison to the P2 cells. These studies indicate unique interactions with both the epidermis and extracellular matrix that are linked to the positional origins of these cells and correlate with their respective response to amputation injury. For example, the interaction between blastema cells and the wound epidermis is essential for a regenerative response following P3 amputation, whereas the contractile activity of the skin is characteristic of a wound healing response at P2 amputations. The differences in interactions with the epidermis and extracellular matrices have obvious ramifications with respect to regenerative competency.

### Regeneration Competence of P2 and P3 cell lines

The two major impediments associated with the generation of regenerative competent cells are the maintenance of competency during expansion in vitro and the ability of these cells to interact within in vivo systems. To ascertain regenerative competence, we began by analyzing the capacity of P3 cells to participate in the regeneration response of the adult digit tip. Amputation midway through the digit tip transects the P3 bone and results in blastema formation and a regeneration response [28] and we have established that connective tissue cells participate in blastema formation (see Fig. 1E, F). The contribution of other cell types present at the amputation injury to the final regenerate have been shown to be largely lineage restricted during regeneration [29], [30], so the current model that emerges is that the blastema is composed of multiple progenitor cell types that proliferate, undergo morphogenesis and re-differentiation without altering their respective developmental fates.

To test the ability of cultured connective tissue cells to participate in blastema formation, we isolated P2 and P3 cells from mice carrying both a GFP marker (*β-actin*-*GFP*) and a LacZ marker (Rosa 26) for cell injection studies. Early passage and stable cell lines were tested by injection into the dorsal region between the nail and the skeletal element at a mid-proximal location of the digit tip of immuno-compromised SCID mice. After a 24 hour healing period the digit tip was amputated midway through the P3 element. In adult mice the digit tip regeneration response involves the formation of a blastema by 10–12 DPA and regenerated bone is evident histologically by 16 DPA [28]. Regenerating digit tips were collected at these time points and analyzed for lacZ positive cell contribution in histological sections. Amputated SCID mice regenerate their digits in parallel with outbred CD1 mice and cell injected amputated digits regenerated normally (not shown).

**Figure 4:**
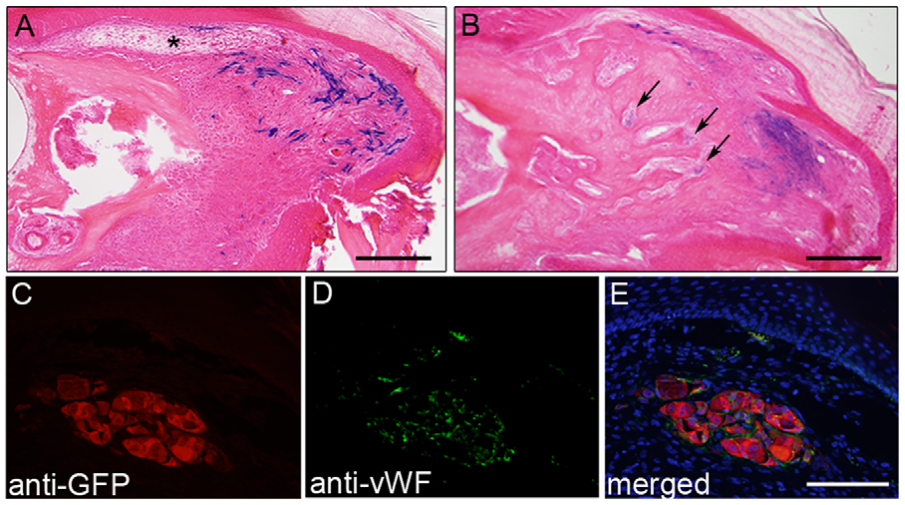
P3 cells are regeneration competent after expansion in vitro. A) LacZ positive P3 cells were injected into the digit tip of SCID mice 1 day prior to amputation and collected at 10 DPA when the regenerate at the blastema stage. LacZ positive cells are present at the injection site in the dorsal connective tissue (*) and are scattered throughout the blastema. B) During the differentiation stage (16 DPA) LacZ positive P3 cells are primarily found in the regenerating connective tissue with small clusters of cells present within the trabeculae of the regenerating bone (arrows). C–E) GFP+ human breast cancer cells injected into P3 connective tissue prior to amputation remained aggregated in the regeneration stump and did not enter the blastema. GFP positive cells (C) shown aggregated in the stump of a 16 DPA regenerate indicating that they survive engraftment. Immunohistochemical co-staining for the endothelial marker vWF (D, E) indicates that these cells differentiate in situ without participating in the regenerative response. A and B, scale bar  = 200 µm; C–E, scale bar  = 100 µm.

The injection of early passage and stable P3 cell lines gave identical results. Many lacZ positive P3 cells were found in the blastema at 10 DPA (n = 3 for early passage; n = 3 for stable cell lines). Some labeled cells were present at the proximally located injection site, however large numbers of labeled cells were found scattered throughout the blastema, including at distal and ventral locations (Fig. 4A). Since the injection site was localized to the dorsal connective tissue of the stump, these observations suggests that P3 cells migrate, or are otherwise trans-located, during the wound healing process that results in blastema formation. Labeled cells found throughout the blastema were contiguous with the injection site suggesting the coordinated movement of labeled cells during the regeneration response. In samples analyzed at 16 DPA when regenerated bone is differentiating proximally (n = 3 for early passage; n = 5 for stable cell lines), labeled cells were largely localized to the connective tissue surrounding the forming bone, however some individual labeled cells were scattered within the forming bone tissue (Fig. 4B). Labeled cells within the bone forming region were localized to the cavities of the trabecular lattice of the regenerating bone and did not appear to differentiate into osteoblasts. These studies demonstrate that P3 cells retain their ability to participate in a regeneration response after isolation and expansion in vitro.

The distribution of P3 cells within the regeneration blastema raised the issue that the blastema itself might be indiscriminately recruiting cells, and that the cell involvement in regeneration may be non-specific. To test this possibility we carried out similar studies using a GFP-labeled human breast cancer cell line (M4A4 LM3-4CL16GFP) that is highly tumorigenic and metastatic in immune compromised mice [50]. This cell line was selected because it is known to survive engraftment, it is a highly invasive cell line that integrates and recruits host cells. Thus, if the blastema microenvironment recruited cells in a non-specific manner, these cells are expected to participate. We found that these cells do not invade or participate in blastema formation, nor to the regeneration response. At 12 DPA (blastema) and 16 DPA (re-differentiation) no GFP- labeled cells were found in the regenerate, instead they formed cell aggregates at the injection site (Fig. 4C) and based on co-immunolocalization studies with anti-VWF we found that the M4A4 LM3-4CL16GFP cells differentiated into endothelial cells (Fig. 4D, E). These studies provide evidence that there is some degree of specificity involved in cellular recruitment to form the regeneration blastema that is retained by cultured P3 cells but is not a general characteristic of highly migratory cells.

**Figure 5:**
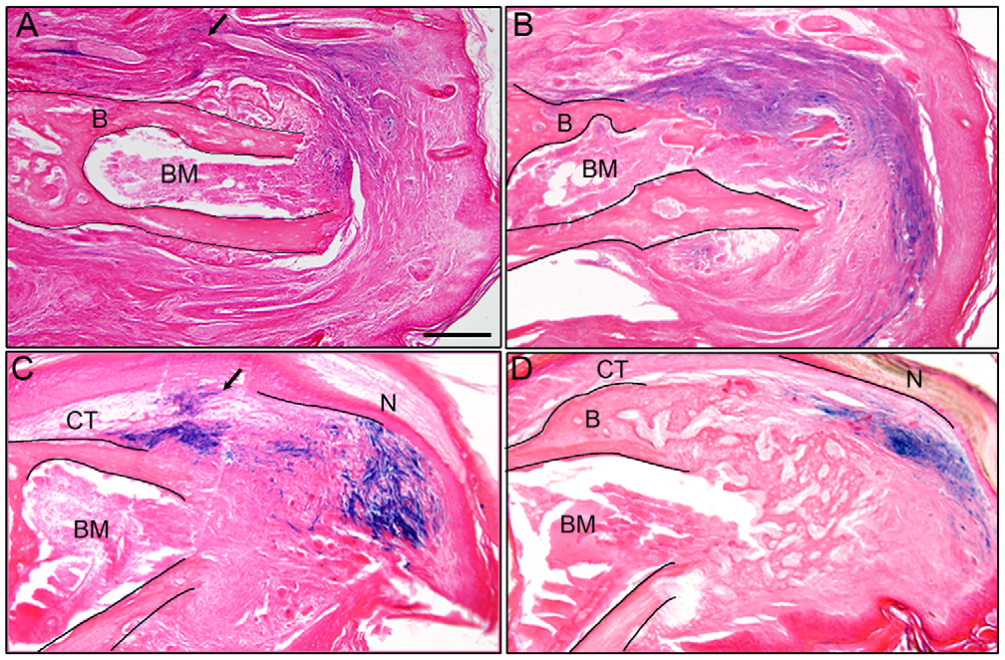
P2 cells participate in a regenerative response. A) Engraftment of LacZ labeled P3 cells into the dorsal P2 region (arrow) prior to amputation shows labeled cells (blue) at 16 DPA participate with non-labeled dermal cells in the wound healing response. B) Control engraftment of LacZ labeled P2 cells into the dorsal P2 region (arrow) prior to amputation shows labeled cells (blue) at 16 DPA also participate with non-labeled dermal cells in the wound healing response. The amputated P2 bone (B) and bone marrow (BM) are outlined in A and B. C) Engraftment of LacZ labeled P2 cells (arrow) into the dorsal P3 connective tissue (CT) prior to digit amputation shows labeled cells (blue) at 10 DPA participating in blastema formation. D) During the differentiation stage (16 DPA) LacZ positive P2 cells (blue) are found in the regenerating connective tissue (CT) and do not associate with the trabeculae of the regenerating bone. The amputated P3 bone (B), bone marrow (BM) and regenerating nail (N) are outlined in the section to demarcate the stump and the regenerate in C and D. A–D, scale bar  = 200 µm.

P2 cells derived from a non-regenerative region of the digit, share some characteristics with P3 cells, however, we have shown that they possess distinct characteristics as well. A key question is whether regeneration competence is a cell autonomous characteristic and/or a characteristic that is regulated by the wound environment. To test if we could rescue regeneration incompetent P2 amputations with P3 cell injections, we injecting labeled P3 cells in to the P2 digit region prior to amputation. We found the P3 injections did not alter the wound healing response and induce regeneration (Fig. 5A), indicating regeneration competence is not cell autonomy. While the P3 cells were able to integrate with the P2 connective tissue, we did not see any differences when compared to control P2 injection into the P2 digit (Fig. 5B). To determine if the P2 cells respond to the regeneration competent environment or induce changes in this environment, we introduced P2 cells into the P3 amputation model in an identical manner to the P3 studies. We tested both early passage P2 cells as well as stable P2 cell lines, and these studies were carried out in parallel with our P3 studies that established regeneration competence. The contribution and distribution of P2 cells in these studies were similar but not identical to our findings for P3 cells. At 10 DPA we found labeled cells at the injection site in the dorsal connective tissue and scattered within the blastema (n = 3) (Fig. 5C). At 16 DPA labeled cells aggregated within the connective tissue as well as the region surrounds the distal regenerated bone (n = 3) (Fig. 5D). Unlike the P3 cells which integrate with the regenerate bone, we observed that the majority of labeled P2 cells were displaced to the regenerating connective tissue and few cells remained associated with the bone. These studies demonstrate that despite their origins from regeneration competent and regeneration incompetent regions of the digit, as well as other differences we characterized in vitro, we find that both P3 and P2 cells are capable of in vitro expansion and participation in blastema formation associated with an epimorphic regeneration response.

### Proliferation and survival during blastema formation

Since P2 and P3 cells displayed clear position-specific characteristics in vitro but both retained the ability to participate in a digit tip regenerative response in vivo, we next explored whether these cells displayed distinct growth and survival characteristics during blastema formation. In these studies we characterized: 1) cell proliferation by analyzing the co-immunolocalization of Ki67 with GFP in engrafted cells, and 2) cell survival by analyzing the co-immunolocalization of cleaved caspase 3 with GFP. These studies focused on the blastema (10 DPA) and compared labeled cells to unlabeled neighboring cells within the same section.

A key aspect of cell engraftment and participation in a regenerative response is assessing the extent to which injected cells integrate into the microenvironment of the host tissue. In non-regenerating control studies we injected P2 or P3 cells into the P3 digit region and analyzed proliferation and survival of cells after 16 days. Cell injections into the adult digit tip without amputation established that both P2 and P3 cells are detected and are integrated into the host connective tissue (not shown). We observed no cleaved caspase 3 positive cells in host connective tissue (n = 12 digits) indicating that any damage to host tissue resulting from the injection procedure itself is rectified within days following microinjection. Similarly, the apoptotic index of GFP labeled P3 (n = 7 digits) and P2 (n = 5 digits) cells in unamputated digits was also found to be zero, indicating these cells successfully engraft and integrate into unamputated host tissue following microinjection.

**Figure 6:**
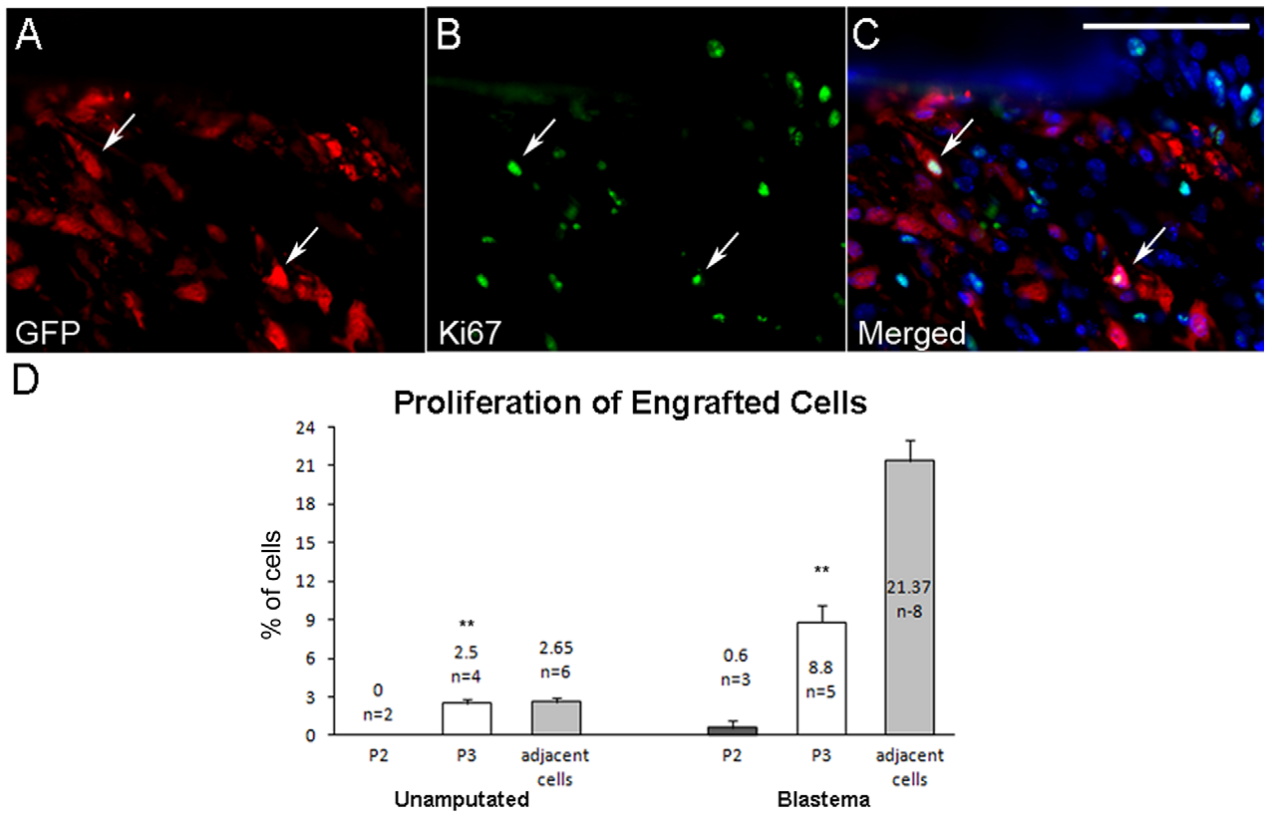
P3 cells proliferate in the blastema. A–C) GFP and Ki67 co-immunohistochemical reveal P3 isolated cells engraft and proliferate. A) GFP labeled P3 cells are identified within the digit. B) Ki-67, a marker for proliferation, is expressed in engrafted and endogenous P3 cells. C) Merged GFP and Ki67 expression identifies a fraction of engrafted P3 cells that are proliferating. Double labeled cells shown in C are indicated with arrows in the image set. GFP- red, Ki67- green, DAPI nuclear stain- blue. Scale bar  = 50 µm. D) Quantification of the proliferation indices of P2 and P3 engrafted cells compared to endogenous neighboring cells. Left: In unamputated digits 17 days post engraftment, P3 cells proliferated at a rate similar to neighboring control cells, whereas P2 cells are non-proliferative. Right: P3 cells participating in blastema formation at 10 DPA display a proliferation index that was lower than neighboring endogenous cells but significantly higher than P2 cells within the blastema. All chart values are expressed as means ± SEM. The proliferative index of P3 cells is significantly greater than P2 cells in both studies (**, p-value <0.005).

We used co-immunostaining with anti-Ki67 and anti-GFP to evaluate cell proliferation following cell engraftment into host tissue (Fig. 6A–C). In the connective tissue of unamputated digits at the P3 level 17 days post-engraftment, the proliferation of endogenous neighboring connective tissue cells was found to be 2.65% (n = 6 digits). Similarly the labeling index of injected P3 cells was 2.5% (n = 4 digits), however P2 cells displayed a much reduced proliferative index following engraftment (0.0%, n = 2 digits). The difference of proliferation index between injected P3 and P2 cells is shown in Figure 6D (left) and was significant based on two-way ANOVA (P<0.05). These data suggested that P3 cells were able to integrate and can adapt to the microenvironment of the P3 connective tissue, whereas P2 cells survive but appeared to be unresponsive to proliferation signals.

We next investigated the participation of P2 and P3 cells in blastema formation by characterizing cell survival and proliferation within the blastema microenvironment. For cell survival we found no evidence of cell death in endogenous blastema cells (n = 12 digits,) indicating that apoptotic cells are not present during the blastema stage of digit tip regeneration. We also failed to find cleaved caspase 3 positive cells among injected P3 cells at 10 DPA (n = 8 digits) indicating a high level of P3 cells survival during the regenerative response. On the other hand, we observed a low frequency of cleaved caspase 3 positive P2 cells at 10 DPA (0.82%, n = 5 digits) that was maintained at later regenerative stages (16 DPA, 1.87%, n = 3 digits). These data indicate that while both P3 and P2 cells successfully engraft into adult connective tissue and can participate in the regenerative response, the regeneration environment favors the survival of P3 cells relative to P2 cells. Since cleaved caspase 3 immunoreactivity of P2 cells was observed at two distinct time points during the regenerative response, it seems likely that there is continuous attrition of P2 cells following amputation injury.

The proliferative response during neonatal and adult digit tip regeneration has been previously described [16], [28]. In the current study the overall proliferation index of endogenous blastema cells at 10 DPA was found to be 21.37% (n = 8 digits). Our analyses of P3 and P2 cells indicate that while both cell lines participate in the formation of the regeneration blastema, they display very different proliferative profiles (Fig. 6D, right). In the 10 DPA blastema, the proliferation index of injected P3 cells was 8.8% (n = 5 digits), whereas the proliferative index of P2 cells was much lower (0.6%, n = 3 digits). These data show that the P3 cells are responsive to proliferation signals present within the regenerating digit blastema, whereas the P2 cells appear to be unresponsive. In summary, while both P3 and P2 cells retain an ability to participate in blastema formation after isolation and expansion in vitro, P3 cells retain a greater capacity to integrate and respond to the blastema microenvironment.

## Discussion

The mouse digit is unique in that the regenerative capabilities of the distal tip appears to have evolved from a non-regenerative state [1], [11], and regeneration from normally non-regenerating digit amputation injury can be induced by extrinsic modification of the amputation wound [24], [51]. One of the key questions for our understanding of mammalian regenerative capability is the role that various cell types play in controlling whether or not a regenerative response is elicited. We have focused on connective tissue cells because fibroblasts have been shown to play a key role in regulating amphibian regeneration [2], and also because transcriptome studies link connective tissue fibroblasts to the maintenance of positional memory in humans [21], [22]. Our study is the first to isolate and characterize cell populations derived from the connective tissue of regeneration-competent (P3) and regeneration-incompetent (P2) regions of the mouse digit. The critical issue addressed by this study relates to our understanding of the regenerative potential of cells that reside in regions of the body that possess regenerative capabilities versus those that fail to regenerate. The most significant finding from these studies is that connective tissue cells from regeneration-incompetent regions are able to participate in a regenerative response, and this conclusion is consistent with recent studies demonstrating enhanced regenerative capabilities of P2 amputations [51]. We also describe position-specific differences in P2 and P3 cells that are suggestive of the maintenance of positional memory after expansion in vitro.

Isolated P2 and P3 cells in vitro are largely similar in a number of characteristics including morphology, proliferative rate, cell surface marker profile, and their expression of selected stem cell and developmental marker genes, yet they display distinct behaviors in organotypic cultures and in their in vivo interaction with blastema cells. In our studies, the number of cells isolated from P2 or P3 connective tissue is small and must be expanded to obtain enough cells for characterization and experimentation. We observe variability in doubling time and spontaneous differentiation in primary cultures that is suggestive of selection in vitro, however once stabilized, our evidence suggests that the cell characteristics during expansion remain largely constant, that the cells display fibroblastic characteristics and retain a “memory” of their position and function. For example, 3-dimensional collagen gel cultures provide a model for *in vivo* wound contraction by characterizing the tension or stress that fibroblasts place on collagen fibers [52], [53]. P3 level amputations resulting in a regenerative response that displays no indication of wound contraction [16], [28], whereas the P2 level amputation undergoes wound contraction followed by a scarring response [19]. Using an in vitro collagen gel stress release model [48], [54], cultured P2 cells develop mechanical tension that contracts the collagen gel, whereas P3 cells lack this ability. Thus, the correlation in in vivo and in vitro behavior is suggestive that connective tissue cells retain characteristics that are appropriate for their position of origin. A similar correlation is observed for the inductive abilities of P2 and P3 cells in controlling the differentiation of keratinocytes in organotypic cultures. Both P2 and P3 cells displayed clearly identified position-dependent characteristics related to epithelial-mesenchymal signaling involved in the control of epidermal differentiation. During the formation of skin in the embryo it is well established that the signals from the presumptive dermis dictate the developmental fate of the overlying ectoderm [55], [56], [57]. In adults there are examples of position-specific interactions in which dermal fibroblasts induce epidermis to differentiate in a site dependent manner [58], [59], [60]. In a similar fashion we find that adult P3 fibroblasts can induce the aggregation of human keratinocytes to form nail-like structures in skin equivalent cultures in vitro, whereas adult P2 cells behave like traditional fibroblasts to induce stratified epidermis in similar cultures. These observations provide evidence that adult P3 cells display a positional memory of their developmental history when they are isolated and expanded in vitro. A similar argument can be made for P2 cells although the characteristic to induce stratified epidermis is much more ubiquitous around the body, and as such it may not be strictly linked to a position effect. Nevertheless, the evidence suggests that isolated and expanded fibroblast progenitor cells maintain a memory of inductive signaling critical for the spatial localization of epidermal characteristics. Since the epidermis is constantly turning over in the adult, this type of positional memory may be important for homeostasis as well as in an injury response.

While the connective tissue associated with phalangeal bones includes a number of different cell types such as fibroblasts, endothelial cells and perivascular cells, our studies suggest that P2 and P3 cells represent fibroblastic cell lines derived from distinct locations along the proximal-distal axis of the digit. The position-specific characteristics displayed by these cells are consistent with human transcriptome studies identifying the fibroblast as the most likely cell type that maintains positional information in adult tissues [21], [22], [61]. By comparison to other well-studied cell lineages that consists of a stem cell that produces progenitor lineages each restricted in their differentiative capacity, such as the hematopoietic lineage [62], the fibroblast lineage appears to maintain a constant phenotype but varies in their ability to regulate position-specific events such as their ability to control keratinocyte differentiation. Whether or not all fibroblasts or a subset of fibroblasts retain or can reacquire positional memory, and how a cell's positional memory changes during a regeneration response remains a critical topic for future studies.

Despite clear differences in the behavior of P2 and P3 cells in vitro, we find that when introduced into an actively regenerating amputation wound, both P3 and P2 cells are able to participate in the formation of a blastema and to contribute to the regenerated digit tip. This leads us to the important conclusion that regeneration-competent cells are present at amputation wounds that fail to regenerate. The important implication from this conclusion is that regenerative failure is not necessarily a consequence of the absence of regeneration-competent cells, but points to an inadequacy of the wound environment to support a regenerative response. This conclusion is also supported by studies showing that modulation of the wound environment with growth factors enhances regenerative responses in neonatal and adult mammals [24], [51], [63], [64]. However, we also find that P2 and P3 cells display distinct growth and survival response within the regenerating P3 blastema: P3 cells survive and are proliferative whereas P2 cells display a low level of cell death and are non-proliferative. This suggests that complex interactions between cells involved in regeneration and, as well, with the wound environment itself play a key role in whether or not a regenerative response is elicited. This conclusion is fundamentally analogous to blastema formation during amphibian limb regeneration where position-dependent interactions between blastema cells within the context of a permissive wound environment govern the regenerative process [65].
